# High coenzyme affinity chimeric amine dehydrogenase based on domain engineering

**DOI:** 10.1186/s40643-022-00528-0

**Published:** 2022-03-27

**Authors:** Jialin Li, Xiaoqing Mu, Tao Wu, Yan Xu

**Affiliations:** 1 Laboratory of Brewing Microbiology and Applied Enzymology, School of Biotechnology, Jiangnan University, Wuxi, 214122 China; 2 Key Laboratory of Industrial Biotechnology, Ministry of Education, School of Biotechnology, Jiangnan University, Wuxi, 214122 China; 3 Suqian Jiangnan University Institute of Industrial Technology, Suqian, 223800 China

**Keywords:** Amine dehydrogenase, Coenzyme affinity, Coenzyme binding domain, Catalytic efficiency

## Abstract

NADH-dependent phenylalanine amine dehydrogenase (F-AmDH) engineered from phenylalanine dehydrogenase (PheDH) catalyzes the synthesis of aromatic chiral amines from prochiral ketone substrates. However, its low coenzyme affinity and catalytic efficiency limit its industrial application. Here, we developed a chimeric amine dehydrogenase, cFLF-AmDH, based on the relative independence of the structure at the domain level, combined with a substrate-binding domain from F-AmDH and a high-affinity cofactor-binding domain from leucine amine dehydrogenase (L-AmDH). The kinetic parameters indicated that cFLF-AmDH showed a twofold improvement in affinity for NADH and a 4.4-fold increase in catalytic efficiency (*k*_cat_/*K*_m_) compared with the parent F-AmDH. Meanwhile, cFLF-AmDH also showed higher thermal stability, with the half-life increased by 60% at 55 °C and a broader substrate spectrum, than the parent F-AmDH. Molecular dynamics simulations suggested that the constructed cFLF-AmDH had a more stable structure than the parent F-AmDH, thereby improving the affinity of the coenzyme. The reaction rate increased by 150% in the reductive amination reaction catalyzed by cFLF-AmDH. When the NAD^+^ concentration was 0.05 mM, the conversion rate was increased by 150%. These results suggest that the chimeric protein by domain shuffling from different domain donors not only increased the cofactor affinity and catalytic efficiency, but also changed the specificity and thermal stability. Our study highlights that domain engineering is another effective method for creating biodiversity with different catalytic properties.

**Graphical Abstract:**

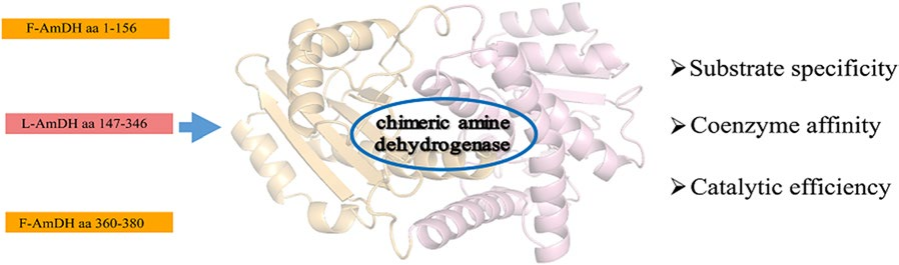

**Supplementary Information:**

The online version contains supplementary material available at 10.1186/s40643-022-00528-0.

## Introduction

Chiral amines are an important class of chiral building blocks that are widely used in fine chemicals, agriculture, biologically active natural products, and pharmaceutical intermediates (Jiang and Fang 2020; Wang and Reetz 2015). Compared with most chemical synthesis pathways that require harsh reaction conditions such as high temperature and high pressure, enzyme catalysis synthesizes chiral chemicals through its high stereo selectivity, mild reaction conditions, and environmental friendliness (Abrahamson et al. 2012; Chen et al. 2018; Sharma et al. 2017). Among the enzymes that have been reported for the synthesis of chiral amines, AmDH only requires cheap ammonium ions as the amino donor, with water as its only by-product, considered a greener synthetic route (Chen et al. 2015; Huang et al. 2016).

At present, there are two main types of AmDH: natural AmDH and engineered AmDH modified from natural amino acid dehydrogenase (Tseliou et al. 2020). However, natural AmDH has few origins, a narrow substrate spectrum, and low catalytic activity, greatly limiting their application in the synthesis of chiral amines (Mayol et al. 2016). Since its creation by Abrahamson et al. (2012) in 2012, worldwide attention has focused on improving the substrate spectrum and increasing the catalytic efficiency by rationally designing a directed evolution-engineered AmDH. Most of the research was aimed at modifying the site near the substrate-binding pocket to improve the properties of AmDH (Ducrot et al. 2020; Grogan 2018; Itoh et al. 2000; Wu et al. 2021). As these are coenzyme-dependent enzymes, reaction efficiency can be improved using a higher coenzyme concentration or increasing the affinity between the enzyme and coenzyme (Franklin et al. 2020; Itoh et al. 2000; Zhou et al. 2019). The latter improves the reaction efficiency and reduces the dosage of expensive coenzymes to reduce production costs. This strategy is gradually being applied (Cai et al. 2020; Jiang and Wang 2020; Li et al. 2019; Wang et al. 2015). In 2021, by alignment with the coenzyme-binding domain sequence of amino acid dehydrogenase which has higher coenzyme affinity, Meng et al. (2021) adjusted the coenzyme-binding cavity increasing the NADH activity of leucine dehydrogenase (LaLeuDH) from *Labrenzia aggregata* by introducing a double mutation in the coenzyme-binding region, thereby increasing reaction efficiency.

With the continuous deepening of research, the modification of AmDH is not limited to directed evolution. Domain shuffling provides an alternative method for obtaining enzymes with improved properties (Kataoka et al. 1994). In 2014, Bommarius et al. (2014) created a novel chimeric amine dehydrogenase (residues 1–149 were contributed by F-AmDH from *Bacillus badius* and 140 to the terminus 366 by the L-AmDH from *Bacillus stereothermophilus*) via domain shuffling, which can catalyze adamantyl methyl ketone to adamantyl ethylamine unlike parent proteins and strongly improve thermal activity. Ch1-AmDH (a chimeric enzyme obtained through domain shuffling of first-generation variants) was used to understand the catalytic mechanism and the molecular discriminants that are crucial for the efficient catalytic activity of AmDHs in 2019 (Tseliou et al. 2019). Crystal structure studies then on AmDHs suggested that the monomer structure of AmDH consists of two independent substrate-binding and cofactor-binding domains (Son et al. 2015a). Subsequently, a chimeric enzyme that changes the specificity of the coenzyme has been reported. By replacing the cofactor NAD^+^ binding domain from *Clostridium symbiotic* to the cofactor NADP^+^ binding domain of glutamate dehydrogenase from *Escherichia coli*, the coenzyme dependency was changed from NADP^+^ to NAD^+^ (Sharkey and Engel, 2009). This chimeric enzyme showed NAD^+^ dependence and high catalytic efficiency. This strategy suggests that the combination or substitution of enzyme domains may contribute to the overall property changes.

Recently, phenylalanine amine dehydrogenases that catalyze difficult aromatic ketone substrates, important precursors of pharmaceutical intermediates, have been gradually reported (Abrahamson et al. 2013; Ruffoni et al. 2019; Zoi et al. 2017). However, their lower coenzyme affinity is limited in industrial production (Kataoka and Tanizawa 2003; Li et al. 2014; Zhu et al. 2016). Here, We report a chimeric enzyme, cFLF-AmDH, based on homologous sequence alignment and structural analysis of the independent substrate and coenzyme of AmDH. The coenzyme-binding domain of L-AmDH from *Bacillus cereus* was used to replace the corresponding region of F-AmDH from *Bacillus badius*, which has a lower coenzyme affinity. The constructed cFLF-AmDH had high coenzyme affinity and catalytic efficiency, and further broadened the substrate spectrum based on inheriting the substrate specificity of F-*Bb*AmDH. Subsequently, we used molecular dynamics (MD) simulations to clarify the factors that help to increase the coenzyme affinity and catalytic efficiency observed in kinetic analysis and conversion experiments. This enables the rational design of coenzyme-binding domains to screen good candidates for improved catalytic efficiency and cofactor affinity.

## Materials and methods

### Strains, plasmids, and chemicals

F-*Bb*AmDH from *Bacillus badius*、L-*Bc*AmDH from *Bacillus cereus* and L-*Es*AmDH from *Exiguobacterium sibiricum* were generated in the laboratory. Glucose dehydrogenase from *Bacillus amyloliquefaciens* (BaGluDH) was purchased from Sigma-Aldrich Corp. (Beijing, China). *Escherichia coli* BL21 (DE3) and plasmid pET-28a (+) were purchased from Novagen (Nanjing, China) as the gene expression host and vector, respectively. p-Fluorophenyl acetone (p-FPA) was bought from J&K Co. Ltd. (Shanghai, China). Isopropyl-*β*-D-thiogalactoside (IPTG), kanamycin, NADH, and NAD^+^ were from TCI (Shanghai, China). All other chemicals were analytical grade and commercially available.

### Construction of cFLF-AmDH expression vectors

The coenzyme-binding domains of two amine dehydrogenases were predicted using the NCBI (https://www.ncbi.nlm.nih.gov). Chimeric amine dehydrogenase (cFLF-AmDH) was introduced using homologous recombination technology containing the coenzyme-binding domain of L-*Bc*AmDH and the substrate-binding domain of F-*Bb*AmDH. The corresponding primers were synthesized by Shenggong Bioengineering (Shanghai) Co., Ltd. (Shanghai China). The PCR program was run for 30 cycles under the following conditions: 30 s at 98 °C, 10 s at 98 °C, 30 s at 55 °C, 70 s at 72 °C, and 10 min at 72 °C, after which it was kept at 10 °C. The constructed plasmids were then isolated and sequenced prior to their introduction into *E. coli* BL21 (DE3). The chimeric strain *E. coli* BL21/pET-28a-cFLFAmDH was obtained after confirmed by DNA sequencing.

### Expression and purification of enzymes

Phenylalanine amine dehydrogenase (F-*Bb*AmDH) was cultured in Luria–Bertani (LB) medium containing kanamycin (50 mg·L^−1^) at 37 °C and with shaking at 200 rpm for 2 h. Protein expression was induced by the addition of IPTG at a final concentration of 0.2 mM when the bacteria reached an OD_600_ value of 0.6–0.8, and the cells were then cultured at 17 °C and 200 rpm for 12 h. cFLF-AmDH was cultured in an auto-induction medium at 37 °C with shaking at 200 rpm for 2 h. When the OD_600_ value of culture was 0.6–0.8, the temperature was adjusted to 17 °C to induce protein expression and cultured for 60 h. The culture was collected by centrifugation at 4 °C and 8000 rpm for 5 min and then stored at − 80 °C until further use. Proteins were expressed in *E. coli* BL21 (DE3) with His_6_-tag at the C-terminus. Cells were lysed by ultrasonic cell crusher and the supernatant was collected by centrifugation at 12,000 × g for 30 min at 4 °C. Proteins were purified with an ÄKTA purifier system (GE Healthcare, Little Chalfont, UK) using Ni–NTA affinity columns and Superdex 200 chromatography. The enzyme purity was determined by SDS-PAGE.

### Kinetic parameter determination

Kinetic parameters of parental F-*Bb*AmDH and cFLF-AmDH were determined in NH_4_Cl/NH_4_OH buffer (100 mM, pH 8.5) at 30 °C in different concentrations of p-FPA (with concentration range from 0.05 to 50 mM) or NADH (with concentration range from 0.01 to 0.5 mM). The kinetic constant (*K*_m_, mmol·L^−1^; *k*_cat_, min^−1^) and catalytic efficiency (*k*_cat_/*K*_m_, L^−1^·min^−1^·mmol^−1^) were calculated using a nonlinear curve fitting of initial velocity versus substrate concentration data to the Michaelis–Menten equation by Origin software. All the values were averaged from three replicates with standard deviations.

### Enzyme activity assay

Enzyme activity was determined by monitoring the change in absorbance at 340 nm at 30 °C using a MultiSkan GO UV-spectrometer (Thermo Fisher Scientific), which corresponds to the change in the concentration of NADH (Li et al. 2014). For reductive amination, the reaction mixture (200 μL) contained 20 mM substrate, 0.2 mM NADH, 2 M NH_4_Cl/NH_4_OH buffer (pH 9.0) and a certain amount of purified enzyme. Enzyme activity unit (U) was defined as the amount of enzyme which catalyzes the production (or consumption) of 1 µmol of NADH per min under the above conditions.

### Enzymatic properties determination

The enzyme activity of F-*Bb*AmDH and cFLF-AmDH was measured at various pH (7.0–11.0) in NH_4_Cl/NH_4_OH buffer (30 °C). The pH stability was determined by enzymatic incubation in NH_4_Cl/NH_4_OH buffer at different pH (7.0–11.0) at 30 °C for 2 h.

The enzyme activity was measured and calculated at various temperatures (30–70 °C) in 2 M NH_4_Cl/NH_4_OH buffer (pH 9.0). Thermal stability of F-*Bb*AmDH and cFLF-AmDH was characterized by half-life. The half-life (t_1/2_) was calculated by incubating in 2 M NH_4_Cl/NH_4_OH buffer (pH 9.0) at 55 °C for different time. The t_1/2_ value at 55 °C was calculated using the following formula: t_1/2_ = ln2 k^−1^ (k is the first-order rate constant, which is derived from the semi-log plot of incubation time and residual activity) (Le et al. 2012). Specific activity before incubation was normalized as 100%.

### Biotransformation and analytical methods

The reaction mixture (10 ml) consisted of 2 M NH_4_Cl/NH_4_OH buffer (pH 8.5), 20 mM NAD^+^, 2 mM P-FBA and 20 μg protein. The reaction was carried out at 37 °C with shaking at 200 rpm for 24 h. Samples (1 mL) were drawn at intervals and kept in boiling water for 10 min to terminate the reaction. The reaction liquid sample (1.0 ml) was extracted twice with 1.0 ml ethyl acetate, and 1.0 ml extract was taken for product determination. The solutions were then passed through a 0.22-μm filter.

The conversion of amine products were performed with GC-FID analysis on a 7890B GC (Agilent) using nitrogen as the carrier gas. Analytic conditions: Grace Econo-Cap EC-WAX + column (30 m × 0.25 mm × 0.25 μm). Split ratio 5:1, pressure 120.0 kPa. Column temperature program: starting at 90 °C, hold for 2 min, with 10 °C·min^−1^ to 180 °C, hold for 2 min.

### Structure modeling, molecular docking, and MD simulation

Amino acid sequence alignment was performed using the MUSCLE server (https://www.ebi.ac.uk/Tools/msa/muscle/) (Madeira et al. 2019) and displayed using the Esprit server (https://espript.ibcp.fr/ESPript/ESPript/) (Robert and Gouet 2014). Protein structure was predicted by Robetta server (https://robetta.bakerlab.org/). Docking of proteins to ligands was obtained with Auto Dock Tools (http://autodock.scripps.edu/resources/adt). Protein structure maps were produced by the 3D visualization software Pymol (https://www.pymol.org). Molecular dynamics (MD) simulations using GROMACS version 5.0.2 and AMBER force field (Ganjoo et al. 2020). System was built in a three-centered water model in an orthogonal box that extends 10 Å from the dissolved atoms in all three dimensions in order to create buffers between them. Use the steepest descent algorithm for energy minimization, a total of 10,000 steps (Son et al. 2015b). In addition, the kinetics of the protein–coenzyme complex and the protein–ligand complex were simulated for 50 ns and the system was heated to 300 K at a pressure of 1.01 bar (Ganjoo et al. 2020). Molecular dynamics simulation trajectories for binding free energy calculations were performed using the MM/GBSA method, defined ΔG_bind_ = G_PL_-G_P_-G_L_ (Genheden and Ryde 2015).

## Results and discussion

### Sequence and structure analysis of parent AmDHs

F-*Bb*AmDH from *Bacillus badius* (Abrahamson et al. 2013) is an engineered amine dehydrogenase that catalyzes aliphatic and aromatic ketone substrates. Compared with other reported engineered amine dehydrogenases, the natural affinity for coenzymes of F*-Bb*AmDH is low, increasing the cost of industrial production. Based on domain recombination technology, coenzyme domain replacement is an effective means of improving parental coenzyme affinity.

Two high-affinity coenzyme domain donors (Table 1), L-*Bc*AmDH from *Bacillus cereus* (Mu et al. 2021) and L-*Es*AmDH from *Exiguobacterium sibiricum* (Chen et al. 2018), were used as donors to obtain a structure similar to that of the amino acid dehydrogenase superfamily (Li et al. 2014). All three enzymes were derived from the introduction to KS/NL double mutations that alter the substrate specificity at the catalytically active center of the corresponding amino acid dehydrogenases. To investigate the structural homology of F-*Bb*AmDH, L-*Bc*AmDH, and L-*Es*AmDH, the amino acid sequences, and tertiary structures were compared using sequence alignment and Robetta server modeling. The whole length of F*-Bb*AmDH had 46.84% and 49.48% amino acid sequence homology with L-*Bc*AmDH and L-*Es*AmDH, respectively (Additional file 1: Figure S1). Meanwhile, F-*Bb*AmDH shared 47.84% and 48.78% amino acid sequence homology with L-*Bc*AmDH and L-*Es*AmDH in the coenzyme-binding region, respectively. The three enzymes were highly similar in their tertiary structure (Additional file 1: Figure S2). L-*Bc*AmDH was chosen as a cofactor domain donor for the higher coenzyme affinity of approximately 2.5-fold compared with L-*Es*AmDH (Table 1).

**Table 1:** Kinetic parameters of the parent for substrate NADH

Enzyme	Substrate	*K*_m_ (mM)	*k*_cat_ (min^−1^)	*k*_cat_/*K*_m_ (mM^−1^ min^−1^)
F-*Bb*AmDH	NADH	0.16 ± 0.02	0.65 ± 0.01	4.03
L-*Bc*AmDH	NADH	0.021 ± 0.004	1.09 ± 0.1	51.40
L-*Es*AmDH	NADH	0.052 ± 0.005	1.62 ± 0.22	31.15

Determination of coenzyme kinetic parameters using the same substrate 2-pentanone. The values were generated by fitting the initial specific activity data to the Michaelis–Menten equation using nonlinear regression with GraphPad Prism software. Value is means ± standard deviations. All reactions involved in the kinetic constant calculations were analyzed using a 2 M NH_4_Cl/NH_4_OH buffer at optimum pH and temperature. All experiments were repeated 3 times

### Coenzyme affinity of chimeric amine dehydrogenase

The structural study suggested that F-*Bb*AmDH is composed of a substrate-binding domain, coenzyme-binding domain, and terminal structure. The chimeric amine dehydrogenase cFLF-AmDH was built from the substrate-binding domain and terminal structure from F-*Bb*AmDH and the coenzyme-binding domain from L-*Bc*AmDH. It was soluble (Additional file 1: Figure S3), expressed by self-induction culture, and the kinetic parameters of the coenzyme were determined according to the Michaelis–Menten equation using nonlinear regression with GraphPad Prism software. Compared with the F-*Bb*AmDH, the *k*_cat_/*K*_m_ values for NADH of cFLF-AmDH increased by 4.2-fold. The *k*_cat_ values were 2.2-fold higher and the *K*_m_ values were twofold lower (Table 2). These results indicated that cFLF-AmDH had a stronger affinity and catalytic efficiency for NADH.

**Table 2:** Kinetic parameters of chimeric amine dehydrogenase and F-*Bb*AmDH for substrate NADH

Enzyme	Substrate	*K*_m_ (mM)	*k*_cat_ (min^−1^)	*k*_cat_/*K*_m_ (mM^−1^ min^−1^)
F-*Bb*AmDH	NADH	0.16 ± 0.02	0.65 ± 0.01	4.03
cFLFAmDH	NADH	0.045 ± 0.005	1.42 ± 0.22	31.56

Comparison of coenzyme kinetics of cFLF-AmDH and F-*Bb*AmDH. Determination of coenzyme kinetic parameters using the same substrate 2-pentanone. The values were generated by fitting the initial specific activity data to the Michaelis–Menten equation using nonlinear regression with GraphPad Prism software. At the same substrate concentration, the kinetic parameters of the coenzyme are measured using NADH of different concentrations, that is, 0.05–0.5 mM

### MD simulation of chimeric amine dehydrogenase-NADH complexes

**Fig. 1:**
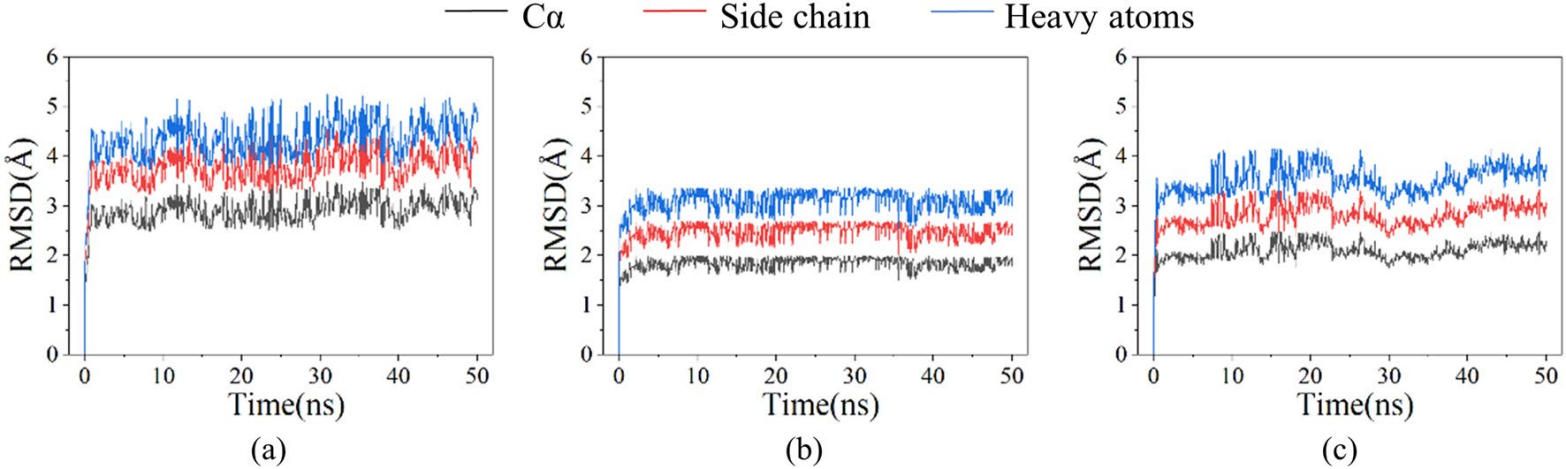
Comparison and analysis of differences between F-*Bb*AmDH, L-*Bc*AmDH and cFLF-AmDH based on MD simulation. The above plot shows the RMSD evolution of F-*Bb*AmDH (**a**), L-*Bc*AmDH (**b**) and cFLF-AmDH (**c**) during the 50-ns simulation at 300 K and 1.01 bar pressure. The first frame is used as the reference. All protein frames are first aligned on the reference frame backbone, and then the RMSD of Cα (black), side chain (red), and heavy atoms (blue) were calculated

To understand the molecular mechanism of the catalytic efficiency improvement of cFLF-AmDH, all-atom MD simulations for F-*Bb*AmDH, L-*Bc*AmDH, and cFLF-AmDH as well as their complexes with NADH at 300 K were performed to analyze the structural changes that occur in proteins. The root-mean-square deviation (RMSD) was used to measure the average deviation of the protein conformation from the original structure (Hyndman and Koehler 2006). As shown in Fig. 1, the RMSD evolution of the complex of F-*Bb*AmDH and NADH showed that the variation range was 2.5–3.5 Å under Cα, 3.0–4.5 Å under the side chain, and 4.0–5.5 Å under the heavy atom. The RMSD evolution of the L-*Bc*AmDH and NADH complexes also showed that Cα, side chains, heavy atoms varied between 1.5–2.0 Å, 2.0–2.5 Å, and 2.5–3.5 Å, respectively. However, the trajectory of the complex of cFLF-AmDH and NADH changed in the range of 1.5–2.5 Å under Cα, 2.5–3.5 Å under the side chain and 3.0–4.5 Å under heavy atoms. For composites with similar structures, the RMSD values and structural stabilities were inversely proportional. The RMSD value of the cFLF-AmDH complex was between F-*Bb*AmDH and L-*Bc*AmDH, which was aligned with the measured catalytic properties. The cFLF-AmDH did not fully characterize the full activity of L-*Bc*AmDH. However, compared with F-*Bb*AmDH, there was a significant change.

### Enzymatic properties of cFLF-AmDH

**Fig. 2:**
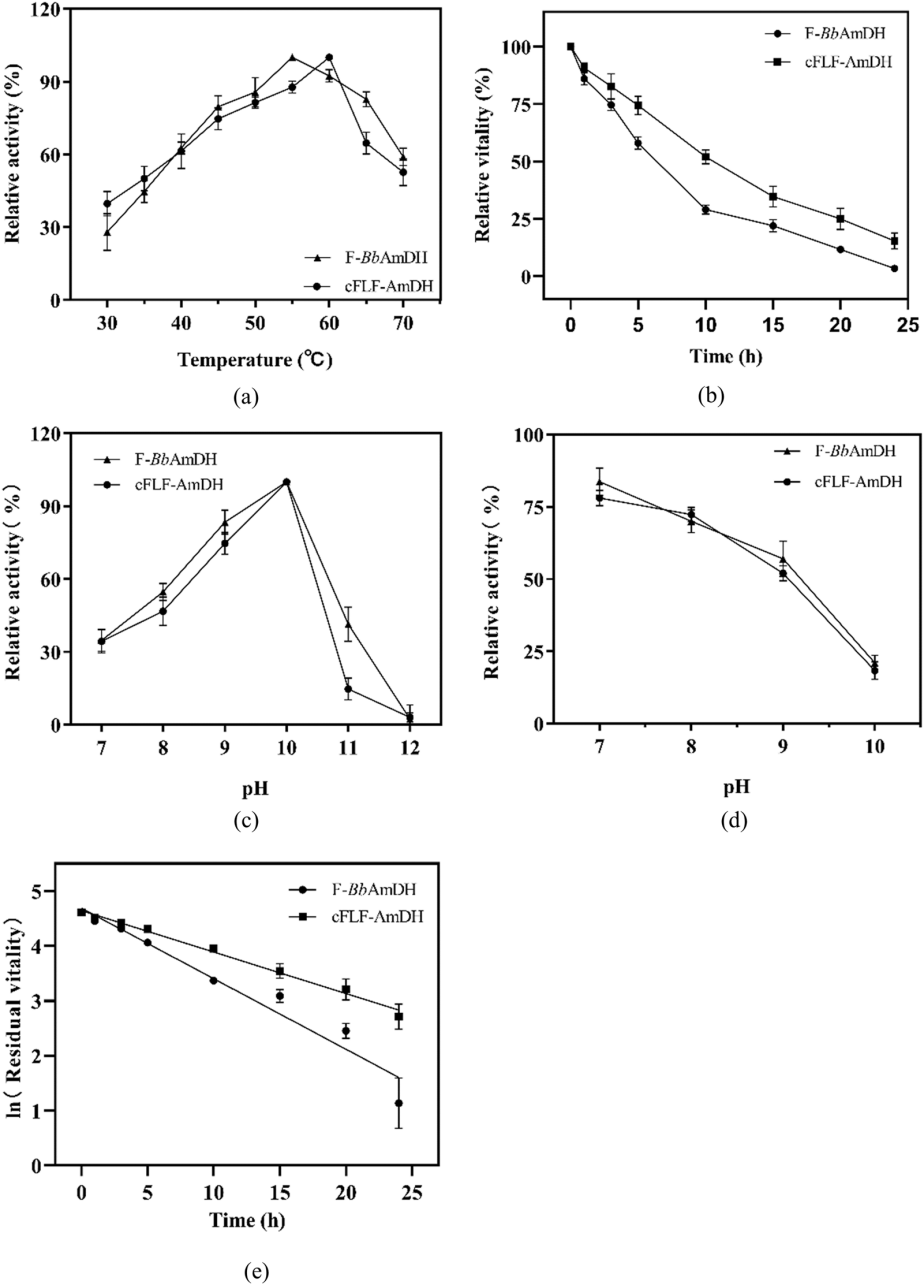
Comparison of enzymatic properties of cFLF-AmDH and F-*Bb*AmDH. **a** Optimum reaction temperature. **b** Temperature stability. **c** Optimum reaction pH. **d** pH stability.** e** Half-life. The error bars showed the standard deviations of three replicates

According to the results of the coenzyme affinity analysis and MD simulation, the chimeric enzyme had better properties. Subsequently, we investigated the enzymatic properties of cFLF-AmDH and F-*Bb*AmDH. The optimal reaction temperatures of cFLF-AmDH and F-*Bb*AmDH were 60 °C and 55 °C, respectively (Fig. 2a). However, thermal optima of cFL1-AmDH and cFL2-AmDH (Bommarius et al. 2014) were both greater than 70 °C. The thermal stabilities researched were carried out at 55 °C by calculating the half-life (*t*_1/2_). The half-life of cFLF-AmDH was 9.6 h which was 160% higher than that of parent F-*Bb*AmDH (6.0 h; Fig. 2b and 2e) and the half-life of cFL1-AmDH (Bommarius et al. 2014) also greater than 500 min at 55 °C.

The optimal reaction pH value and stability of F-*Bb*AmDH and cFLF-AmDH were similar (Fig. 2c and 2d). Although the optimal reaction pH value was 10.0, the residual activity after 2 h of incubation decreased as the pH value increased. It retained more than 80% and only 20–25% activity at a neutral pH (7.0) and alkaline pH (10.0), respectively.

### Substrate specificity of cFLF-AmDH

Structurally, part of the amino acid residues in the coenzyme-binding domain will participate in the formation of the substrate-binding pocket, so the replacement of the coenzyme-binding domain may cause changes in substrate specificity and activity. The activities of a series of aliphatic ketone substrates and aromatic ketone substrates of cFLF-AmDH and F-*Bb*AmDH were investigated and compared. For both aliphatic (Table 3) and aromatic (Table 4) ketone substrates, cFLF-AmDH had higher catalytic activity and broad substrate specificity than F-*Bb*AmDH. The activity of cFLF-AmDH to 2-pentanone, p-fluorophenyl acetone (p-FPA), 4-methylpropiophenone, and p-methoxypropiophenone increased by 500%, 250%, 200%, and 170%, respectively. cFLF-AmDH accepted new substrates such as 4-methyl-2-pentanone, 5-hydroxy-2-pentanone, acetophenone, and 3-methylacetophenone. Our cFLF-AmDH had activity against acetophenone and pFPA at 30 °C, however, cFL1-AmDH was hardly active at 30 °C (Bommarius et al. 2014).

**Table 3:** Specific activity (mU mg^−1^) of F-*Bb*AmDH and cFLF-AmDH toward aliphatic ketones

Substrates	Specific activity (mU mg^−1^)^a^	
	F-*Bb*AmDH	cFLF-AmDH
4-Methyl-2-butanone	n.a.^b^	n.a
4-Hydroxy-2-butanone	n.a	n.a
2-Pentanone	33.5	143.32
4-Methyl-2-pentanone	n.a	25.29
5-Hydroxy-2-pentanone	n.a	34.75

^a^Activity was measured in NH_4_Cl/NH_4_OH buffer (2 M, pH 9.0) containing 0.2 mM NADH and 20 mM substrate at 30 °C

^b^n.a. = no measurable activity

**Table 4:** Specific activity (mU mg^−1^) of F-*Bb*AmDH and cFLF-AmDH toward aromatic ketones

Substrates	Specific activity (mU mg^−1^)^a^	
	F-*Bb*AmDH	cFLF-AmDH
Acetophenone	n.a.^b^	21.96
2-Methylacetophenone	n.a	n.a
3-Methylacetophenone	n.a	23.32
4-Methylacetophenone	n.a	n.a
4-Fluoroacetophenone	n.a	n.a
3-Hydroxyacetophenone	n.a	n.a
4-Methoxyacetophenone	n.a	n.a
4-Fluorophenylacetone	719.8	1759.4
4-Methylpropiophenone	377.9	838.1
4-Hydroxypropiophenone	n.a	n.a
P-Methoxyphenylacetone	199.1	337.9
4-(4-Methoxyphenyl)-2-butanone	n.a	n.a

^a^Activity was measured in NH_4_Cl/NH_4_OH buffer (2 M, pH 9.0) containing 0.2 mM NADH and 20 mM substrate at 30 °C

^b^n.a. = no measurable activity

p-FPA was selected as the test substrate because phenylacetone, the simplest ketone analog of phenylpyruvate, the natural substance of phenylalanine dehydrogenase, was not readily available as a controlled substance and its vitality increased the most in this study. To observe the changes in coenzyme affinity and substrate affinity, the kinetic parameters were determined with pFPA and NADH at various concentrations. The obtained kinetic data are summarized in Table 5. cFLF-AmDH yielded 1.7-fold lower *K*_m_ values and 1.2-fold increases in *k*_cat_ values. This led to a 2.1-fold increase in *k*_cat_/*K*_m_ values for NADH. Compared with F-*Bb*AmDH, cFLF-AmDH gave threefold higher *k*_cat_ values and 2.6-fold higher *K*_m_ values for pFPA reduction, resulting in a 1.2-fold increase in the *k*_cat_/*K*_m_ values. These results indicated that the affinity of cFLF-AmDH for coenzymes increased and that the catalytic efficiency of the coenzyme and substrate pFPA improved. For comparison with cFL1-AmDH and cFL2-AmDH, we also measured coenzyme kinetic parameters at 60 °C. The *K*_m_ and *K*_cat_ values of cFLF-AmDH were 0.05 mM and 132.44 min^−1^, respectively, at 60 °C. Although the *K*_m_ value was not much different, the *K*_cat_ value of cFLF-AmDH was 2.5-fold larger than that of cFL1-AmDH (Bommarius et al. 2014).

**Table 5:** Kinetic parameters of the F-*Bb*AmDH and cFLF-AmDH

Enzyme	pFPA			NADH		
	*K*_m_ (mM)	*k*_cat_ (min^−1^)	*k*_cat_/*K*_m_ (min^−1^ mM^−1^)	*K*_m_ (mM)	*k*_cat_ (min^−1^)	*k*_cat_/*K*_m_ (min^−1^ mM^−1^)
F-*Bb*AmDH	8.262 ± 1.13	56.74 ± 4.83	6.60	0.86 ± 0.05	47.66 ± 3.46	55.40
cFLF-AmDH	21.81 ± 2.67	173.4 ± 6.94	7.95	0.49 ± 0.02	56.91 ± 5.12	116.14

The values were generated by fitting the initial specific activity data to the Michaelis–Menten equation using nonlinear regression with GraphPad Prism software. Value is means ± standard deviations. All reactions involved in the kinetic constant calculations were analyzed using a 2 M NH_4_Cl/NH_4_OH buffer at optimum pH and temperature. All experiments were repeated 3 times

**Fig. 3:**
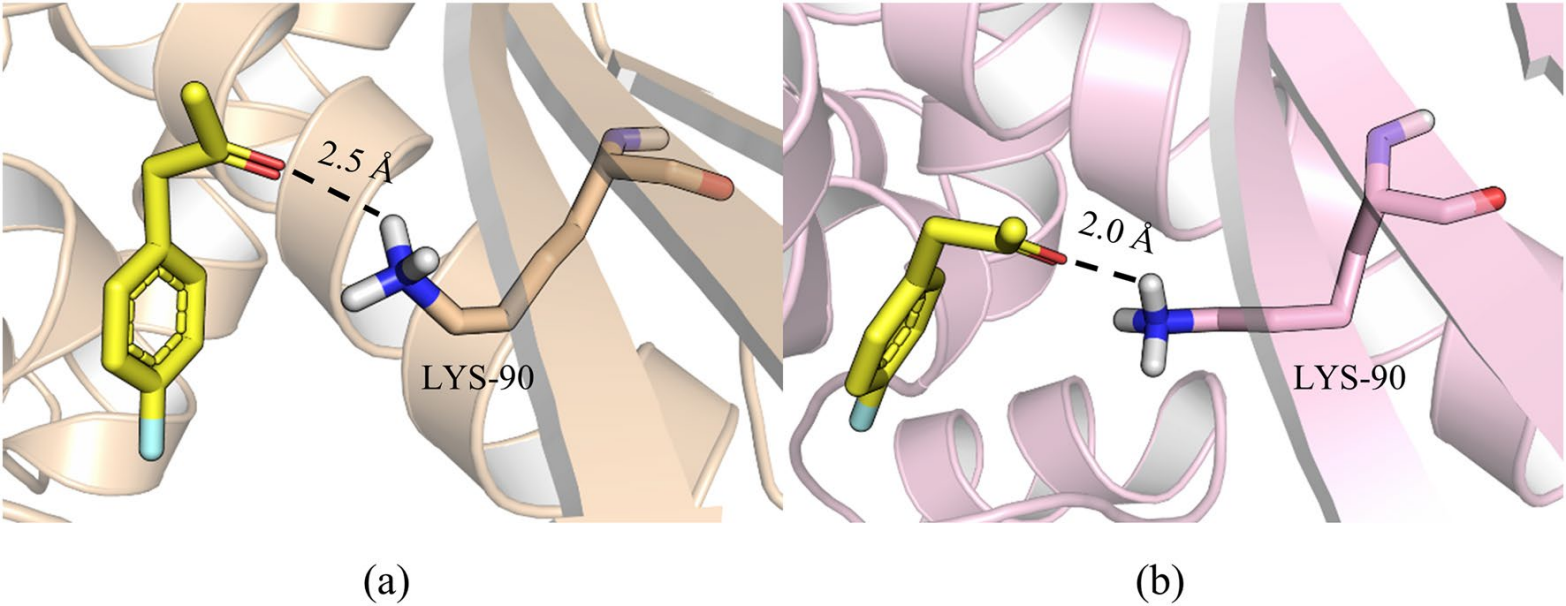
Molecular docking of pFPA with F-*Bb*AmDH (**a**) and cFLF-AmDH (**b**). Substrate pFPA is shown as yellow sticks. Key residue K90 is shown as cyan sticks

**Fig. 4:**
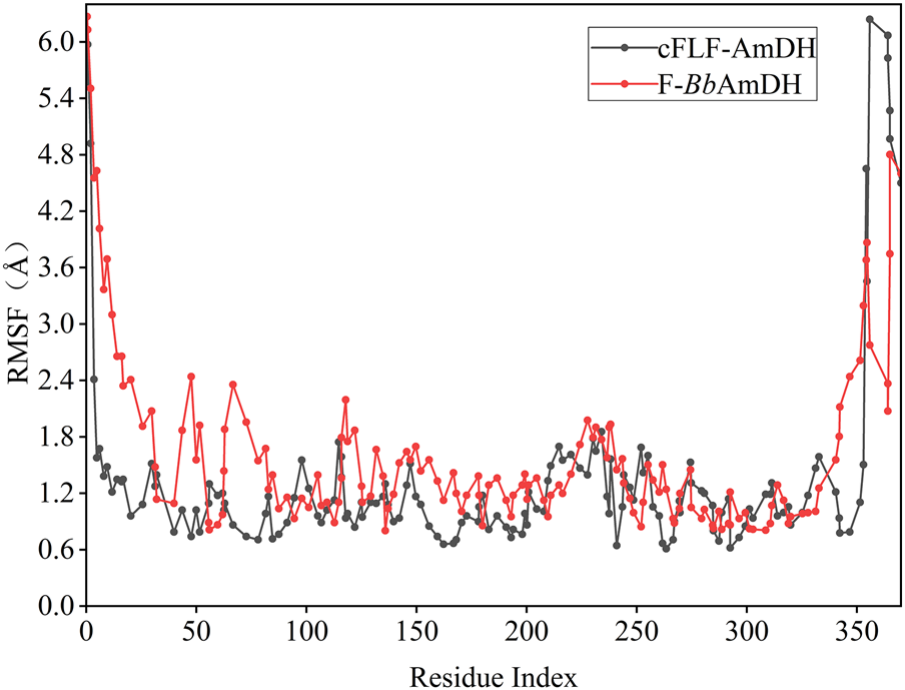
Comparison and analysis of differences between cFLF-AmDH and F-*Bb*AmDH based on MD simulation. The RMSF results of cFLF-AmDH (black) and F-*Bb*AmDH (red) are shown in the line chart. They were calculated for all frames in the trajectory

In the docking analysis, the distance between p-FPA and the side chain of cFLF-AmDH-K90 was 2.0 Å. The K90 was the key residue for the interaction between ammonia and the substrate (Fig. 3). MD simulation studies the structural changes that occur when a substrate binds to a protein (Fig. 4). The root-mean-square fluctuation (RMSF) is useful for characterizing local changes along the protein chain. According to the RMSF value, the structure of cFLF-AmDH was more stable. The cFLF-AmDH model can be used as a promising template to produce chiral amines through a semi-rational design. Meanwhile, the aliphatic ketone substrate 2-pentanone and the aromatic substrate pFPA were selected as representative substrates and calculated binding energy. The results are shown in Table 6. Lower energy requirements for binding to cFLF-AmDH whether it was aliphatic ketone or aromatic substrates.

**Table 6:** The binding energies (Δ*G*_bind_) of substrates and enzyme

Substrates	ΔG_bind_(kcal/mol)	
	F-*Bb*AmDH	cFLF-AmDH
pFPA	− 5.9	− 8.2
2-Pentanone	− 4.8	− 5.5

### Reduction reactions of cFLF-AmDH with different coenzyme concentrations

**Fig. 5:**
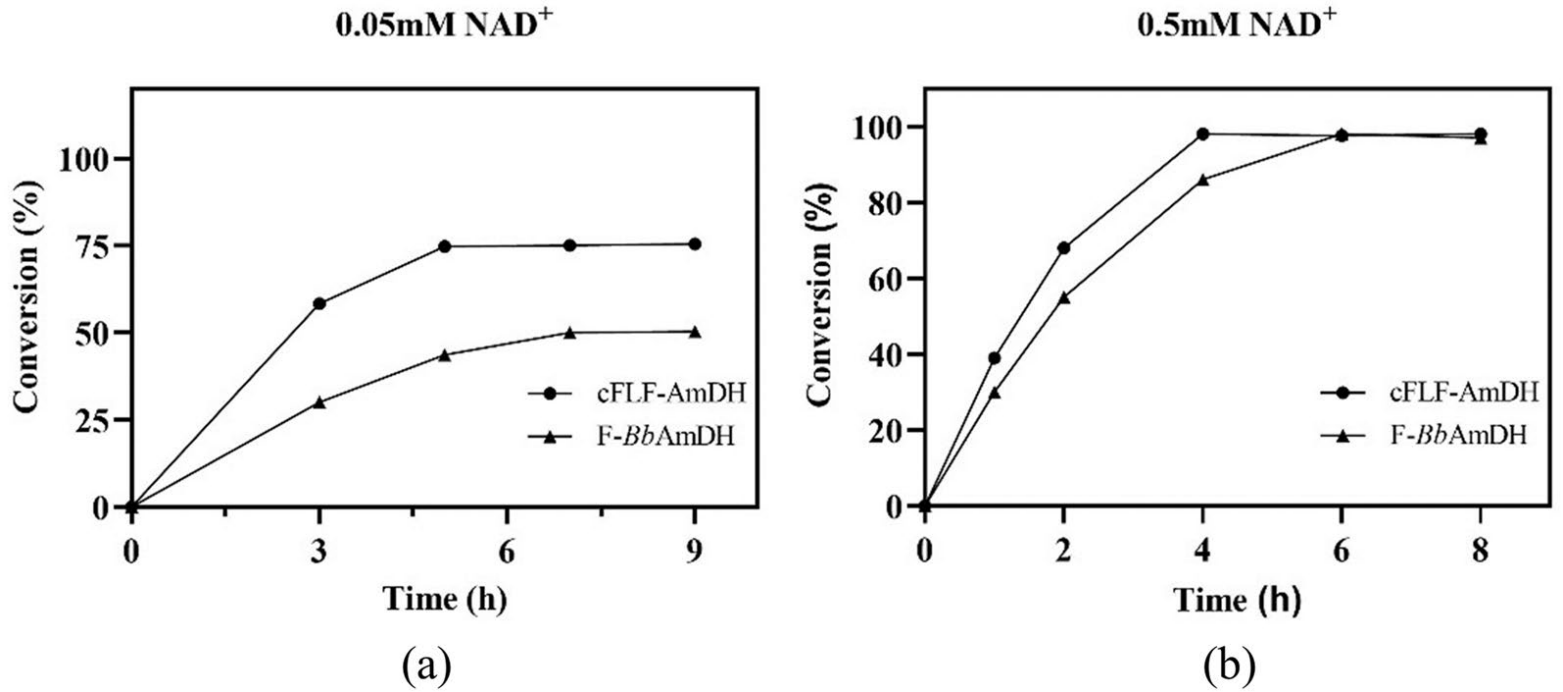
Effect of NAD^+^ concentration on the reductive amination of pFPA. **a** 0.05 mM NAD^+^; **b** 0.5 mM NAD^+^. Reaction conditions: pFPA (0.2 M), NH_4_Cl–NH_3_H_2_O (2 M), glucose (0.24 M) and purified cFLF-AmDH and F-*Bb*AmDH. (1 gL^−1^) in Tris–HCl buffer (0.1 M, pH 8.5), 30 °C, 200 rpm

Chimeric amine dehydrogenase cFLF-AmDH showed higher catalytic activity (*k*_cat_/*K*_m_) for coenzyme affinity. Consequently, the reductive amination reaction of cFLF-AmDH was carried out at a coenzyme concentration of 0.05 mM and 0.5 mM with p-FPA as substrate. The reaction curves are shown in Fig. 5; the maximum reaction conversion reached 75% catalyzed by cFLF-AmDH, whereas, it only reached 50% catalyzed by parent F-AmDH with 0.05 mM of NAD^+^. When the coenzyme concentration increased to 0.5 mM, it took about 4 h for cFLF-AmDH to reach the 1 conversion of 100%, whereas, it took about 6 h for F-*Bb*AmDH. The reaction results agreed with the previous dynamic parameter results of cFLF-AmDH for NADH. Thus, by increasing the catalytic efficiency of the enzyme for the coenzyme, the reaction proceeded more efficiently.

## Conclusion

In summary, to reduce the cost of industrial applications of biocatalysts and to improve the utilization efficiency of enzymes for coenzymes, we rationally designed and created a high NADH-affinity chimeric amine dehydrogenase cFLF-AmDH using coenzyme-binding structural analysis. This led to a decrease in *K*_m_ of the enzyme for NADH from 0.86 to 0.49 mM, with the reaction time shortened from 6 to 4 h. To gain deeper insight, MD simulation analysis was used to explore the binding relationship between the coenzymes and enzymes in substrate catalysis. The results showed that the chimeric amine dehydrogenase cFLF-AmDH we constructed had a more stable structure and shortened distance between the key residue sites for substrate binding. These findings provide a good basis for the industrial application of this enzyme. The strategy employed in this study can also be used to discover other enzymes with specific functions and to improve the efficiency of coenzyme utilization by oxidoreductases.

### Supplementary Information

**Additional file 1: Figure S1**. Sequence Alignment. Amino acid sequence alignment of F-*Bb*AmDH, L-*Bc*AmDH, L-*Es*AmDH, and *Bs*LeuDH. Alignment was performed using the MUSCLE server (https://www.ebi.ac.uk/Tools/msa/muscle/) and displayed using Esprit (http://espript.ibcp.fr). Secondary structure elements are shown based on the *Bs*LeuDH structure. Protein structure is predicted by Robetta server (https://robetta.bakerlab.org/). **Figure S2.** Structure comparison. Docking of proteins to ligands was obtained with Auto Dock Tools (http://autodock.scripps.edu/resources/adt). Protein structure maps were produced by the 3D visualization software Pymol (https://www.pymol.org). FBbAmDH is shown as green cartoon, L-BcAmDH is shown as orange cartoon, LEsAmDH is shown as pink cartoon. **Figure S3.** SDS-PAGE analysis the cell-free extract of cFLFAmDH using LB medium (**A**), SDS-PAGE analysis the cell-free extract of cFLFAmDH using autoinduction medium (**B**). A: M, molecular weight marker, Lane 1 ~ 2, the cell-free extract of cFLFAmDH, Lane 3 ~ 4, broken centrifugal sediment of cFLFAmDH. B: M, molecular weight marker, Lane 1 ~ 3, the cell-free extract of cFLFAmDH, Lane 4 ~ 6, purified enzymes of cFLFAmDH.

## Data Availability

All data generated or analyzed during this study are included in this published article and its supplementary information files.

## Abbreviations

F-AmDH: Phenylalanine amine dehydrogenase
cFLF-AmDH: Chimeric amine dehydrogenase
L-AmDH: Leucine amine dehydrogenase
pFPA: P-Fluorophenylacetone
