# Supplementary material for: High coenzyme affinity chimeric amine dehydrogenase based on domain engineering
Source: Bioresour Bioprocess. 2022 Mar 27;9(1):33. doi: 10.1186/s40643-022-00528-0 (PMC10992376; doi:10.1186/s40643-022-00528-0)
Supplement: Supplementary file 1 — Additional file 1: Figure S1. Sequence Alignment. Amino acid sequence alignment of F-BbAmDH, L-BcAmDH, L-EsAmDH, and BsLeuDH. Alignment was performed using the MUSCLE server (https://www.ebi.ac.uk/Tools/msa/muscle/) and displayed using Esprit (http://espript.ibcp.fr). Secondary structure elements are shown based on the BsLeuDH structure. Protein structure is predicted by Robetta server (https://robetta.bakerlab.org/). Figure S2. Structure comparison. Docking of proteins to ligands was obtained with Auto Dock Tools (http://autodock.scripps.edu/resources/adt). Protein structure maps were produced by the 3D visualization software Pymol (https://www.pymol.org). FBbAmDH is shown as green cartoon, L-BcAmDH is shown as orange cartoon, LEsAmDH is shown as pink cartoon. Figure S3. SDS-PAGE analysis the cell-free extract of cFLFAmDH using LB medium (A), SDS-PAGE analysis the cell-free extract of cFLFAmDH using autoinduction medium (B). A: M, molecular weight marker, Lane 1 ~ 2, the cell-free extract of cFLFAmDH, Lane 3 ~ 4, broken centrifugal sediment of cFLFAmDH. B: M, molecular weight marker, Lane 1 ~ 3, the cell-free extract of cFLFAmDH, Lane 4 ~ 6, purified enzymes of cFLFAmDH. [file 40643_2022_528_MOESM1_ESM.docx]

**Supporting information**

# Highly coenzyme affinity chimeric amine dehydrogenase based on domain engineering

Jialin Li^1,2,3‡^, Xiaoqing Mu^1,2,3‡,*^, Tao Wu^1,2^, and Yan Xu^1,2^

^1^Laboratory of Brewing Microbiology and Applied Enzymology, School of Biotechnology, Jiangnan University, Wuxi 214122, China

^2^Key Laboratory of Industrial Biotechnology, Ministry of Education, School of Biotechnology, Jiangnan University, Wuxi 214122, China

^3^Suqian Jiangnan University Institute of Industrial Technology, Suqian 223800, China

^‡^These authors contributed equally

*^*^*Corresponding Author: xqmu@jiangnan.edu.cn (X. M)

**
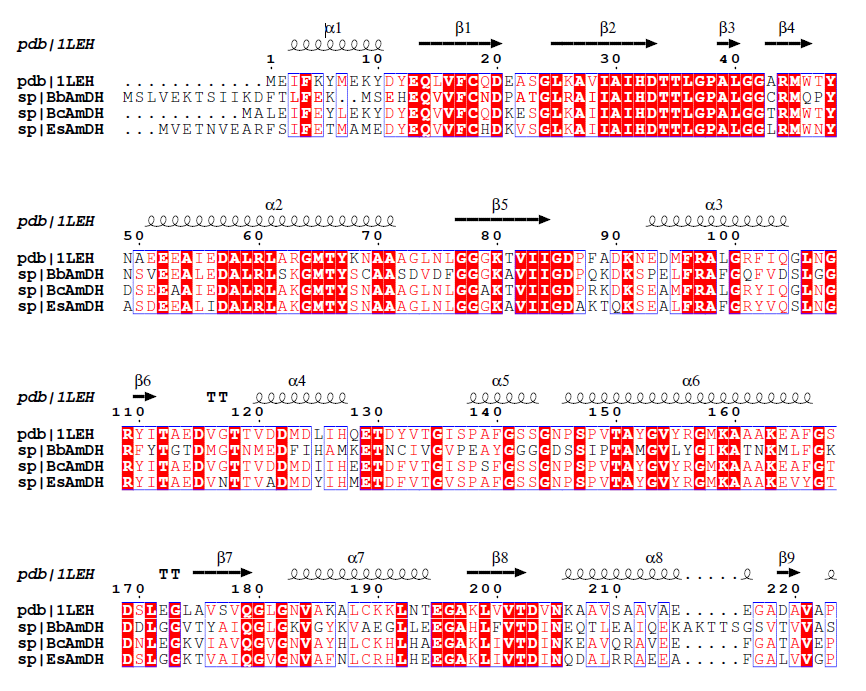
**

**
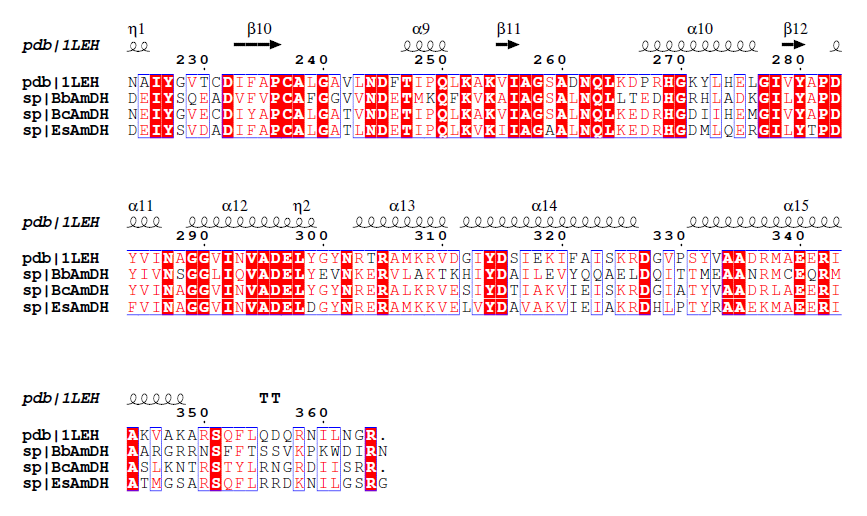
**

**Figure S1.** Sequence Alignment. Amino acid sequence alignment of F-*Bb*AmDH, L-*Bc*AmDH, L-*Es*AmDH, and *Bs*LeuDH. Alignment was performed using the MUSCLE server (https://www.ebi.ac.uk/Tools/msa/muscle/) and displayed using Esprit (http://espript.ibcp.fr). Secondary structure elements are shown based on the *Bs*LeuDH structure. Protein structure is predicted by Robetta server (https://robetta.bakerlab.org/).

**
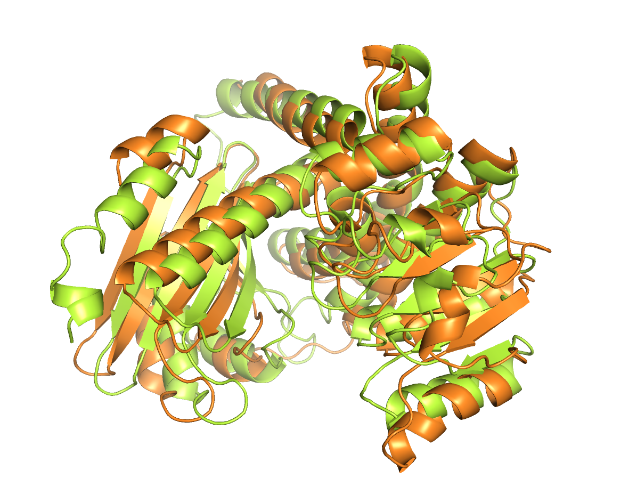

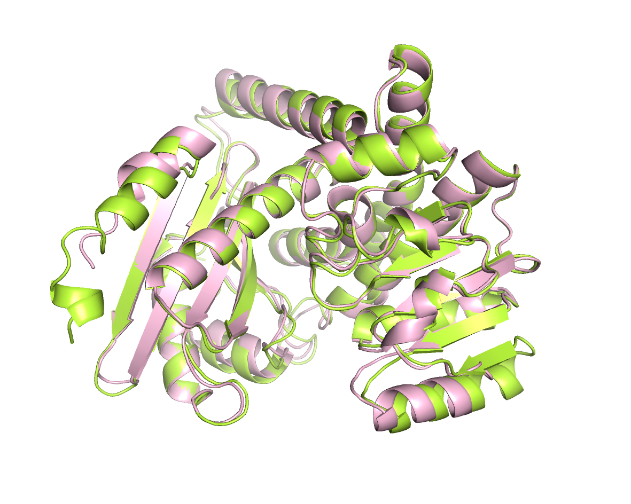
**

**Figure S2.** Structure comparison. Docking of proteins to ligands was obtained with Auto Dock Tools (http://autodock.scripps.edu/resources/adt). Protein structure maps were produced by the 3D visualization software Pymol (https://www.pymol.org). F-*Bb*AmDH is shown as green cartoon, L-*Bc*AmDH is shown as orange cartoon, L-*Es*AmDH is shown as pink cartoon.

**
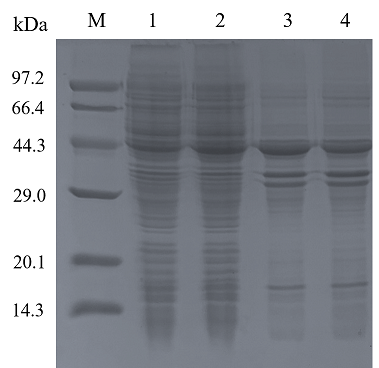

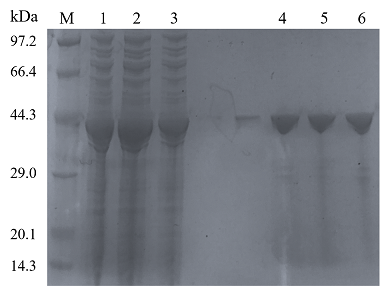
**

1. (B)

**Figure S3.** SDS-PAGE analysis the cell-free extract of cFLFAmDH using LB medium (A), SDS-PAGE analysis the cell-free extract of cFLFAmDH using auto-induction medium (B). A: M, molecular weight marker, Lane 1 ~ 2, the cell-free extract of cFLFAmDH, Lane 3 ~ 4, broken centrifugal sediment of cFLFAmDH. B: M, molecular weight marker, Lane 1 ~ 3, the cell-free extract of cFLFAmDH, Lane 4 ~ 6, purified enzymes of cFLFAmDH.

**Enzymes sequences**

**>F-*Bb*AmDH**

ATGTCGTTGGTGGAAAAAACTTCAATCATTAAGGATTTCACACTGTTCGAAAAGATGAGTGAACATGAGCAAGTTGTCTTTTGCAATGATCCCGCAACTGGTTTACGCGCGATCATCGCTATCCATGATACCACTTTAGGCCCAGCACTTGGAGGATGTCGCATGCAGCCTTACAATAGTGTCGAAGAGGCATTGGAGGACGCGTTGCGCTTGTCGAAAGGCATGACATATGGATGTGCGGCCTCTGACGTGGACTTTGGCGGCGGCAAGGCAGTGATTATTGGTGATCCCCAAAAGGACAAGTCCCCGGAGCTGTTTCGCGCATTTGGTCAGTTCGTGGATAGCCTTGGAGGACGTTTTTACACGGGCACAGATATGGGGACCAATATGGAAGACTTTATTCACGCAATGAAGGAGACGAATTGCATCGTCGGAGTACCGGAGGCTTATGGAGGTGGAGGCGACTCGTCTATTCCGACTGCCATGGGGGTCCTGTATGGAATCAAGGCAACCAATAAGATGTTGTTCGGTAAGGATGATTTAGGCGGTGTTACCTACGCGATCCAGGGCCTTGGTAAGGTGGGGTACAAAGTTGCCGAAGGTTTACTTGAAGAAGGGGCACATTTGTTTGTAACCGATATCAACGAACAAACTTTAGAAGCCATCCAAGAGAAGGCAAAGACTACTTCCGGCTCCGTGACCGTGGTGGCGTCCGACGAAATTTACTCGCAGGAAGCAGATGTGTTCGTTCCCTGTGCTTTCGGAGGTGTCGTGAACGACGAAACCATGAAGCAGTTTAAAGTAAAGGCGATTGCTGGGTCAGCGCTGAATCAGTTATTAACCGAGGACCATGGCCGCCACCTGGCCGACAAAGGGATTCTGTATGCGCCCGACTACATTGTTAATTCAGGAGGCCTTATCCAAGTAGCTGACGAATTGTATGAAGTGAACAAGGAACGTGTGCTTGCAAAGACAAAACATATCTATGACGCTATCTTAGAAGTATACCAGCAGGCTGAATTGGATCAGATTACAACCATGGAAGCTGCTAATCGCATGTGCGAACAGCGTATGGCAGCGCGTGGTCGCCGTAACTCCTTCTTTACGTCTAGTGTGAAGCCGAAATGGGACATTCGTAATTAA

**>L-*Bc*AmDH**

ATGGCATTAGAAATCTTCGAATACTTAGAAAAATATGATTATGAGCAAGTAGTATTTTGTCAAGATAAAGAATCAGGTTTAAAAGCAATTATTGCAATTCATGATACAACACTTGGACCGGCTCTTGGTGGAACAAGAATGTGGACATATGATTCTGAAGAAGCGGCGATTGAAGATGCATTGCGTCTTGCAAAAGGGATGACATACTCTAACGCAGCAGCTGGTTTAAACTTAGGTGGTGCAAAAACAGTAATTATCGGTGATCCACGTAAAGATAAGAGCGAAGCAATGTTCCGTGCGTTAGGCCGTTACATTCAAGGATTAAACGGACGTTACATTACAGCTGAAGATGTTGGTACAACTGTAGATGATATGGATATTATCCACGAAGAAACTGACTTTGTAACAGGGATTTCACCATCATTCGGTTCTTCTGGTAACCCATCTCCAGTAACTGCATACGGTGTTTACCGTGGTATGAAAGCAGCTGCAAAAGAAGCTTTCGGTACTGATAATTTAGAAGGAAAAGTAATTGCTGTTCAAGGTGTTGGTAACGTAGCATATCACCTATGCAAACATTTACACGCTGAAGGAGCAAAATTAATCGTTACAGATATTAATAAAGAAGCTGTACAACGTGCGGTAGAAGAATTTGGTGCGACAGCTGTTGAACCAAATGAGATTTACGGTGTTGAATGTGATATTTACGCACCATGTGCATTAGGCGCAACAGTAAATGATGAAACTATTCCACAACTTAAAGCAAAAGTAATCGCAGGTTCTGCACTGAACCAATTAAAAGAAGATCGTCACGGCGACATCATTCATGAAATGGGTATTGTATACGCGCCAGACTATGTTATTAATGCAGGTGGCGTAATTAACGTAGCAGACGAGTTATATGGATATAATAGAGAACGTGCATTAAAACGCGTTGAGTCAATTTATGACACAATTGCAAAAGTAATCGAAATTTCAAAACGCGATGGCATTGCAACTTATGTAGCAGCAGATCGTCTAGCTGAAGAGCGCATTGCAAGCTTGAAAAACACTCGTAGCACATACTTACGCAACGGTCGCGACATTATTAGCCGTCGCTAA

**>L-*Es*AmDH**

ATGGTTGAAACAAACGTAGAAGCACGATTCAGTATTTTCGAAACGATGGCAATGGAAGATTACGAACAAGTCGTATTTTGTCACGATAAAGTCTCAGGATTAAAGGCGATTATCGCGATTCATGATACGACACTCGGACCAGCACTCGGCGGACTCCGTATGTGGAACTATGCGTCTGACGAGGAAGCATTGATCGACGCGCTTCGTTTGGCAAAAGGCATGACGTATTCTAATGCGGCAGCCGGTCTGAACCTTGGCGGCGGGAAAGCGGTCATCATCGGTGATGCGAAAACGCAAAAATCAGAAGCTCTGTTCCGTGCATTCGGTCGTTACGTACAGTCGTTAAACGGACGTTACATCACTGCGGAAGACGTCAACACAACAGTCGCCGACATGGATTATATCCACATGGAAACAGACTTCGTAACCGGTGTCAGCCCGGCATTCGGATCAAGCGGCAATCCGTCACCAGTCACGGCTTATGGCGTTTACCGCGGAATGAAGGCAGCCGCTAAAGAAGTATATGGCACAGATTCACTCGGAGGAAAAACAGTTGCGATTCAAGGTGTTGGTAACGTTGCTTTCAACCTATGCCGTCACTTGCATGAAGAAGGCGCAAAATTGATTGTCACAGACATCAATCAAGATGCATTACGCCGTGCAGAAGAAGCGTTTGGCGCTCTCGTCGTCGGACCGGATGAAATTTACAGCGTCGATGCCGATATCTTTGCGCCGTGTGCCTTAGGTGCGACATTGAACGATGAGACGATTCCACAACTGAAAGTGAAAATCATTGCCGGAGCAGCACTCAACCAACTCAAAGAAGATCGTCACGGAGATATGCTCCAGGAACGCGGTATTTTATATACACCGGACTTCGTCATCAACGCAGGAGGTGTCATCAATGTGGCCGACGAACTCGACGGGTACAACCGTGAGCGGGCGATGAAAAAAGTCGAACTCGTCTATGATGCGGTAGCAAAAGTCATCGAAATTGCCAAACGTGACCATCTGCCGACTTACCGGGCAGCAGAGAAGATGGCAGAAGAACGGATCGCGACAATGGGCAGTGCCCGCAGCCAGTTCTTACGCCGGGATAAAAACATTTTAGGATCACGCGGTTAA
